# Sensitivity of epidermal growth factor receptor and ErbB2 exon 20 insertion mutants to Hsp90 inhibition

**DOI:** 10.1038/sj.bjc.6603950

**Published:** 2007-08-21

**Authors:** W Xu, S Soga, K Beebe, M-J Lee, Y S Kim, J Trepel, L Neckers

**Affiliations:** 1 Urologic Oncology Branch, Center for Cancer Research, National Cancer Institute, Bethesda, MD, USA; 2 Medical Oncology Branch, Center for Cancer Research, National Cancer Institute, Bethesda, MD, USA

**Keywords:** Hsp90, epidermal growth factor receptor mutation, ErbB2 mutation, kinase inhibition, non-small cell lung cancer

## Abstract

The mature epidermal growth factor receptor (EGFR) neither associates with nor requires the molecular chaperone heat-shock protein 90 (Hsp90). Mutations in EGFR exons 18, 19, and 21 confer Hsp90 chaperone dependence. In non-small cell lung cancer (NSCLC), these mutations are associated with enhanced sensitivity to EGFR inhibitors *in vitro* and with clinical response *in vivo*. Although less prevalent, insertions in EGFR exon 20 have also been described in NSCLC. These mutations, however, confer resistance to EGFR inhibitors. In NSCLC, exon 20 insertions have also been identified in the EGFR family member ErbB2. Here, we examined the sensitivity of exon 20 insertion mutants to an Hsp90 inhibitor currently in the clinic. Our data demonstrate that both EGFR and ErbB2 exon 20 insertion mutants retain dependence on Hsp90 for stability and downstream-signalling capability, and remain highly sensitive to Hsp90 inhibition. Use of Hsp90 inhibitors should be considered in NSCLC harbouring exon 20 insertions in either EGFR or ErbB2.

Both ErbB2 and epidermal growth factor receptor (EGFR) have distinct requirements for heat-shock protein 90 (Hsp90). While the nascent forms of both proteins require interaction with the chaperone for proper maturation, only ErbB2 retains dependence on Hsp90 at the plasma membrane (Xu *et al*, 2001, 2002). However, Shimamura *et al* (2005) recently reported that certain EGFR kinase domain mutations which occur in a sub-population of non-small cell lung cancer (NSCLC) and which enhance sensitivity to EGFR inhibitors, also conferred dependence on Hsp90 and resulted in acquired sensitivity of the mature protein to Hsp90 inhibition. These mutations include deletions in exon 19, and single amino-acid substitutions in exons 18 and 21 (Lynch *et al*, 2004; Paez *et al*, 2004; Tsao *et al*, 2005). A smaller percentage of EGFR mutations found in NSCLC (∼9%) are insertions in exon 20 of EGFR (Kosaka *et al*, 2004; Mitsudomi *et al*, 2005; Shigematsu *et al*, 2005a; Tam *et al*, 2006). Of 1108 NSCLC patients examined, EGFR exon 20 insertions were identified in 1.6% (Greulich *et al*, 2005). Importantly, however, in contrast to mutations in exons 18, 19, and 21, exon 20 insertions render the EGFR resistant to inhibitors such as gefitinib and erlotinib (Greulich *et al*, 2005). Likewise, exon 20 insertions in the EGFR family member ErbB2 (found in ∼2% of NSCLC) confer resistance to these drugs (Wang *et al*, 2006), making it imperative to identify alternative strategies to inhibit the activity and signalling capability of these ErbB mutants (Shimamura *et al*, 2006). To this end, we examined the Hsp90 association and dependence of several ErbB (EGFR and ErbB2) exon 20 insertion mutants that have been identified in NSCLC.

## MATERIALS AND METHODS

### Cells, plasmid treatments, and western blotting

COS7 cells were maintained in a humidified atmosphere containing 5% CO_2_ in DMEM with 10% fetal bovine serum and antibiotics. Exon 20 insertion mutations in EGFR were created by using the QuickChange XL mutagenesis kit following the manufacturer's protocol (Stratagene, La Jolla, CA, USA). The primers used: for EGFR/M766InsASV are CCTACGTGATGGCCAGCGTGGCCAGCGTGGACAAC, reversed and complemented; for EGFR/N771InsPH are GATGGCCAGCGTGGACAACCCCCACCCCCACGTGTGCCGCCTGC, reversed and complemented; for EGFR/D770InsNPH are GGCCAGCGTGGACAACCCCCACAACCCCCACGT, reversed and complemented; for EGFR/D770InsNPG are GGCCAGCGTGGACAACCCCGGCAACCCCCACGT, reversed and complemented; and for EGFR/V738InsKIPVAI are GAACCTGAGAAAGTTAAAATTCCCGTCGCTATCAAAATTCCCGTCGC, reversed and complemented. COS7 cells were transfected with mutant EGFR constructs using FuGene 6 (Roche Diagnostics, Indianapolis, IN, USA) and following the manufacturer's instructions. One day after transfection, cells were treated with/without 0.5 *μ*M 17-AAG (a kind gift from Kosan Biosciences, Hayward, CA, USA) for 1 h, and lysed with TMNS (50 mM Tris–HCl, pH 7.0, 20 mM Na_2_MoO_4_, 0.09% NP-40, 150 mM NaCl) buffer supplemented with 20 *μ*g ml^−1^ aprotinin, 20 *μ*g ml^−1^ leupeptin, and 1 mM PMSF. Epidermal growth factor receptor proteins were immunoprecipitated with Ab-13 antibody (Lab Vision, Freemont, CA, USA), separated by 4–20% gradient SDS–PAGE, and transferred to PVDF (polyvinylidene fluoride) membranes, which were probed sequentially for Hsp90 and EGFR.

To examine effects of 17-AAG on stability, autophosphorylation, and downstream signalling of EGFR exon 20 insertion mutants, we transfected COS7 cells as indicated. Two days after transfection, cells were treated with 0.5 *μ*M 17-AAG, 0.1 *μ*M gefitinib, or a combination of the two drugs for 5 h. Cells were lysed with modified RIPA buffer (50 mM Tris, pH 7.5, 1% NP40, 0.5% sodium deoxycholate, 150 mM NaCl, 1 mM EDTA, 2 mM sodium orthovanadate, 2 mM NaF) supplemented with 8 mM sodium orthovanadate and protease inhibitors. Equivalent amounts of protein were separated by 4–20% gradient SDS–PAGE and transferred to nitrocellulose. Individual membranes were probed as indicated.

ErbB2 exon 20 insertion mutations were prepared using the method described above. Primers used: for ErbB2/A771InsYVMA are GAAGCATACGTGATGGCTTACGTGATGGCTGGTGTGGGCTCCCCATATG, reversed and complemented; for ErbB2/V777InsGSP are GCTGGTGTGGGCTCCCCAGGCTCCCCATATGTCTCCCGCCTTCTGG, reversed and complemented. COS7 cells were transfected as above with mutant ErbB2 constructs. One day after transfection, cells were treated with/without 0.5 *μ*M 17-AAG for 1 h, and lysed with TMNS buffer supplemented with 20 *μ*g ml^−1^ aprotinin, 20 *μ*g ml^−1^ leupeptin, and 1 mM PMSF. The ErbB2 proteins were immunoprecipitated with Ab-5 antibody (Oncogene) and processed as for EGFR analysis. In some cases, COS7 cells were treated for 4 h two days after transfection with 1 *μ*M 17-AAG. Cells were lysed with modified RIPA buffer supplemented with 2 mM sodium orthovanadate and protease inhibitors. Equivalent amounts of protein were separated by 4–20% gradient SDS–PAGE and transferred to nitrocellulose. Individual membranes were probed as indicated.

The NCI-H1781 NSCLC cells, harbouring a G776InsV_G/C in exon 20 of ErbB2, were treated as above and association of Hsp90 before and after 17-AAG was monitored. The effect of 17-AAG on the viability of NCI-H1781 cells was determined by monitoring trypan blue exclusion on successive days using a Cellometer AutoT4 (Nexcelom Bioscience, Lawrence, MA, USA). Determinations were made in duplicate and data are expressed as a mean percentage of viable cells in treated *vs* untreated cells at each time point ±s.d.

## RESULTS AND DISCUSSION

**Figure 1:**
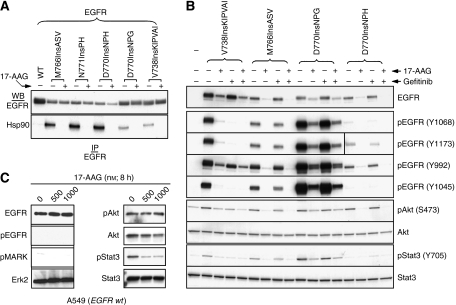
Exon 20 insertion mutations in EGFR confer interaction with and dependence on Hsp90. (**A**) Epidermal growth factor receptor exon 20 insertion mutants display 17-AAG-sensitive association with Hsp90. COS7 cells were transfected with wild type or mutant EGFR constructs as described in Materials and Methods. One day after transfection, cells were treated with/without 0.5 *μ*M 17-AAG for 1 h, and Hsp90 association with EGFR was monitored by coimmunoprecipitation analysis. Membranes were probed sequentially for Hsp90 and EGFR as shown. (**B**) Stability, autophosphorylation, and downstream signalling of EGFR exon 20 insertion mutants are inhibited by 17-AAG. Two days after transfection, COS7 cells were treated with 0.5 *μ*M 17-AAG, 0.1 *μ*M gefitinib, or a combination of the two drugs for 5 h. Cells were lysed and processed as described in Materials and Methods. Individual membranes were probed as indicated. The EGFR phosphorylation sites examined form docking sites linking the activated receptor to various downstream effectors: EGFR/pY1068 associates with Grb2, Stat3/5, and Gab1; EGFR/pY1173 associates with Shp1 and Ptp1; EGFR/pY992 associates with PLC*γ* and Shc; and EGFR/pY1045 associates with Cbl. (**C**) A549 cells, expressing wild-type EGFR, were treated with 17-AAG as shown. Cells were lysed and processed as described in Materials and Methods, and Western blots were probed as in (**B**) above.

To date, no existing NSCLC cell line has been identified to naturally express EGFR exon 20 insertion mutations. Therefore, to study the Hsp90-dependence of EGFR exon 20 insertion mutants we transiently transfected into COS7 cells four mutant EGFRs (V738InsKIPVAI, M766InsASV, D770InsNPG, D770InsNPH) that have been identified in tumour tissue of NSCLC patients. Twenty-four hours later, cells were treated with 17-AAG, an Hsp90 inhibitor currently in multiple phase II clinical trials. In contrast to transiently transfected wild-type EGFR, Hsp90 could be readily coimmunoprecipitated with each of the mutant EGFRs, and this association was remarkably sensitive to brief (1 h) exposure of cells to 17-AAG (0.5 *μ*M) (Figure 1A). Failure to detect Hsp90 association with mature wild-type EGFR in this experiment is consistent with our earlier observation that, unlike mature ErbB2, wild-type EGFR is resistant to Hsp90 inhibitor-induced degradation (Xu *et al*, 2001, 2005).

Next, we examined the effects of gefitinib and 17-AAG, separately and together, on EGFR activation status and on constitutive activation of the prosurvival Akt and Stat3 downstream-signalling pathways, previously shown to be key mediators of mutant EGFR-dependent tumorigenesis (Greulich *et al*, 2005; Jing and Tweardy, 2005; Uchida *et al*, 2007). Although not all exon 20 insertion mutants were equally active, their expression in COS7 cells led to equivalent upregulation of the steady-state phosphorylation of endogenous Akt and/or Stat3-signalling proteins (Figure 1B). EGFR/D770insNPG, the most active mutant, was insensitive to gefitinib (0.1 *μ*M, 5 h), as were the less active mutants EGFR/D770InsNPH and EGFR/M766InsASV. Surprisingly, the activity of EGFR/V738InsKIPVAI was inhibited by gefitinib, as was the activation status of both downstream effectors Akt and Stat3. However, all four EGFR insertion mutants were quite sensitive to 17-AAG (0.5 *μ*M, 5 h), as the steady-state level of both total and phosphorylated EGFR declined markedly after Hsp90 inhibitor treatment. The uniform downregulation of all autophosphorylated docking sites in EGFR suggests that Hsp90 inhibition effectively uncouples the kinase from its various downstream-signalling pathways (Schulze *et al*, 2005). In support of this hypothesis, 17-AAG inhibited the activation of Akt and Stat3 independent of the particular EGFR insertion mutant under examination, and with the possible exception of EGFR/V738InsKIPVAI, its activity did not appear to be additive with that of gefitinib. For comparison, the effect of 17-AAG on wild-type EGFR and several downstream-signalling pathways is shown in Figure 1C. Stat3 phosphorylation remains sensitive to 17-AAG, even in the absence of wild-type EGFR downregulation. From these data, we conclude that EGFR exon 20 insertion mutants, even though they resist inhibition by gefinitib, depend strongly on association with Hsp90 for their stability and function. Brief exposure to a clinically achievable concentration of 17-AAG promoted rapid downregulation of their activity and the activity of two important downstream-signalling pathways.

**Figure 2:**
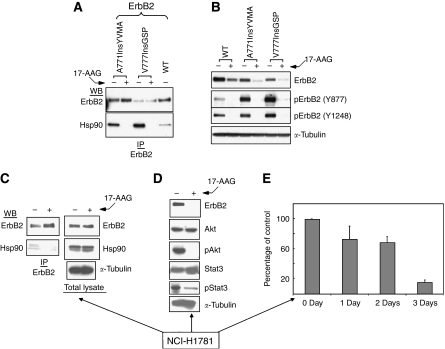
Exon 20 insertion mutations in ErbB2 do not abrogate association with or dependence on Hsp90. (**A**) ErbB2 exon 20 insertion mutants retain 17-AAG-sensitive association with Hsp90. The COS7 cells were transfected with wild-type or mutant ErbB2 constructs as shown. One day after transfection, cells were treated with/without 0.5 *μ*M 17-AAG for 1 h, and lysed as above. The appearance of Hsp90 in ErbB2 immunoprecipitates was examined. (**B**) ErbB2 exon 20 insertion mutants retain sensitivity to Hsp90 inhibition. Two days after transfection, COS7cells were treated with 1 *μ*M 17-AAG for 4 h. Cells were lysed and processed as described. Equivalent amounts of protein were separated by 4–20% gradient SDS–PAGE and transferred to nitrocellulose. Individual membranes were probed as indicated. (**C**) NCI-H1781 NSCLC cells, harbouring a G776InsV_G/C in exon 20 of ErbB2, were treated as above and association of Hsp90 before and after 17-AAG were monitored. (**D**) The effect of 17-AAG (0.5 *μ*M) on downstream signalling (phospho-Akt and phospho-Stat3) in NCI-H1781 cells was examined as described above. (**E**) 17-AAG (0.5 *μ*M) promotes time-dependent loss of viability of NCI-H1781 cells. Determinations were made in duplicate and data are expressed as a mean percentage of viable cells in treated *vs* untreated cells at each time point ±s.d.

Unlike EGFR, mature wild-type ErbB2 depends on Hsp90 association for stability and, among client proteins of the chaperone, ErbB2 is perhaps the most sensitive to Hsp90 inhibition (Chavany *et al*, 1996; Mimnaugh *et al*, 1996). Although both ErbB2 and EGFR are highly homologous proteins, distinct surface characteristics of a short loop in the N-lobe of the kinase domain of both proteins determine Hsp90 binding and sensitivity to 17-AAG (Xu *et al*, 2005). The ErbB2 exon 20 mutations result in constitutive phosphorylation and activation of both ErbB2 and EGFR and they confer resistance to clinically approved EGFR tyrosine kinase inhibitors (Wang *et al*, 2006). As the amino acids comprising this loop are encoded by nucleotides in exon 20, it was of particular interest to determine if NSCLC ErbB2 exon 20 insertion mutants (found in ∼2% of NSCLC) acquired independence from Hsp90 and resistance to 17-AAG. We examined three naturally occurring ErbB2 insertion mutants (Stephens *et al*, 2004; Shigematsu *et al*, 2005b; Buttitta *et al*, 2006; Willmore-Payne *et al*, 2006). Plasmids expressing either wild-type ErbB2, or the insertion mutants ErbB2/A776InsYMVA or ErbB2/V777InsGSP (not found in NSCLC cell lines) were transfected into COS7 cells and their association with Hsp90 was examined (Figure 2A). Unexpectedly, the two insertion mutants associated more strongly with Hsp90 than with wild-type ErbB2, but in each case brief exposure (1 h) to 17-AAG disrupted association with the chaperone. Functional dependence of these insertion mutants on Hsp90 was further supported by the marked sensitivity of total and phospho-ErbB2 to slightly longer exposure (4 h) to 17-AAG (Figure 2B). The NSCLC cell line NCI-H1781 naturally expresses an ErbB2 protein carrying a G776InsV_G/C mutation in exon 20 (Shimamura *et al*, 2006; a mutation previously identified in a number of NSCLC adenocarcinomas). Endogenous mutant ErbB2 in NCI-H1781 coimmunoprecipitated with Hsp90 and this association was disrupted by 17-AAG (Figure 2C). Further, analogous to our earlier data examining downstream signalling from EGFR exon 20 insertion mutants, 17-AAG strongly suppressed Akt- and Stat3-signalling pathways in NCI-H1781 cells, concomitant with loss of ErbB2 protein (Figure 2D). Finally, we observed a time-dependent loss of cell viability following exposure of these cells to 17-AAG (Figure 2E); this is the expected outcome if NCI-H1781 cells were ‘addicted’ to this ErbB2 insertion mutant, as has been previously suggested (Shimamura *et al*, 2006).

Non-small cell lung cancer expressing EGFR and ErbB2 exon 20 insertion mutants have proven resistant to currently approved EGFR tyrosine kinase inhibitors. Preliminary clinical data suggest that 17-AAG, which is well-tolerated *in vivo*, displays activity in the context of ErbB2-positive metastatic breast cancer (Modi *et al*, 2005). Further, Shimamura *et al* (2005) have shown recently that EGFR exon 19 deletion mutants, exons 18 and 21 substitution mutants, and exon 20 T790M point mutants all require Hsp90 and are sensitive to chaperone inhibition. In light of these data and our current findings, we suggest that 17-AAG, or other Hsp90 inhibitor, should be considered as a viable therapeutic alternative in all instances of EGFR and ErbB2 kinase domain mutation, including the 3–4% of NSCLC that express exon 20 insertions in either of these proteins.
